# GLP-1 receptor agonists are associated with reduced nicotine dependence in hidradenitis suppurativa: A TriNetX new-user, active-comparator study

**DOI:** 10.1016/j.jdin.2026.04.006

**Published:** 2026-04-14

**Authors:** Nuran C. Golbasi, Shari R. Lipner

**Affiliations:** a Weill Cornell Medicine, New York, New York; b Department of Dermatology, Weill Cornell Medicine, New York, New York

**Keywords:** Cox proportional hazards model, GLP-1 receptor agonists, hidradenitis suppurativa, nicotine dependence, retrospective, substance use disorders, TriNetX

*To the Editor:* Hidradenitis suppurativa (HS) is a chronic inflammatory skin disease associated with metabolic dysfunction and tobacco use. Glucagon-like peptide-1 (GLP-1) receptor agonists, prescribed for common HS comorbidities such as obesity and diabetes, have been implicated in addictive behaviors, including nicotine dependence.^1^ However, this relationship has not been examined in HS, where smoking is strongly linked to disease risk and outcomes.^2^^,^^3^ Therefore, we performed a new-user, active-comparator cohort study using a large multicenter database.

TriNetX is a research network with patient data from >100 healthcare organizations that applies internal quality checks. On December 29, 2025, TriNetX was queried for patients with HS newly prescribed GLP-1 agonists versus metformin from 2005 to 2025, with index date defined as treatment initiation. Metformin served as an active comparator to reduce confounding by indication. Patients with prior substance use were excluded, and a 365-day washout period without exposure to study medications was required. Propensity score-matching balanced pre-index demographics, psychiatric and cardiometabolic comorbidities, HS-related treatments, and healthcare utilization. Cox proportional hazards models estimated hazard ratios (HRs) of nicotine dependence and other substance use disorders.

After matching, baseline characteristics were well-balanced (all SMDs <0.10; Table I). Over 3-years, patients with HS initiated on GLP-1 agonists vs metformin initiators had reduced incidence of nicotine dependence (3.37% vs 4.28%; HR = 0.73; 95% CI: 0.62-0.86; *P* < .001) and other substance use disorders including alcohol, cannabis, cocaine, and stimulants (1.62% vs 2.04%; HR = 0.74; 95% CI: 0.58-0.93; *P* = .011), both remaining significant after Bonferroni correction (Table II). In a 5-year sensitivity analysis, results remained consistent.

**Table I:** Demographic and clinical characteristics in patients with HS initiating GLP-1 agonists vs metformin after matching

Characteristics	GLP-1 agonists (*N* = 7544)	Metformin (*N* = 7544)	SMD
Demographics			
Age, y	43.5 ± 13.7	43.2 ± 15.6	0.017
Female sex	6490 (86.0)	6477 (85.9)	0.005
White race	3761 (49.9)	3783 (50.1)	0.006
Black race	2648 (35.1)	2635 (34.9)	0.004
Asian race	151 (2.0)	144 (1.9)	0.007
Hispanic ethnicity	846 (11.2)	873 (11.6)	0.011
Comorbidities			
Overweight and obesity	5277 (69.9)	5281 (70.0)	0.001
Hypertension	3173 (42.1)	3168 (42.0)	0.001
Type 2 diabetes mellitus	2254 (29.9)	2283 (30.3)	0.008
Dyslipidemia	2761 (36.6)	2715 (36.0)	0.013
Chronic kidney disease	378 (5.0)	328 (4.3)	0.031
Anxiety disorders	3023 (40.1)	2984 (39.6)	0.011
Depressive disorders	2425 (32.1)	2389 (31.7)	0.010
HS-related treatments			
Incision and drainage procedures	931 (12.3)	913 (12.1)	0.007
Systemic glucocorticoids	5123 (67.9)	5072 (67.2)	0.014
Systemic antibiotics	3464 (45.9)	3440 (45.6)	0.006
Immunosuppressants	1021 (13.5)	1005 (13.3)	0.006
Adalimumab	502 (6.7)	517 (6.9)	0.008
Healthcare utilization			
Outpatient visits	4602 (61.0)	4565 (60.5)	0.010

Values are shown as *n* (%) for categorical variables or mean ± standard deviation (SD) for continuous variables. All post-match SMDs <0.10 indicate well-balanced covariates. Mean follow-up was 682 ± 373 days in the GLP-1 agonist cohort and 634 ± 402 days in the metformin cohort. ICD-10, ATC, RxNorm, and CPT codes used to define comorbidities, medications, and procedures are listed in Supplementary Table I, available via Mendeley at https://data.mendeley.com/datasets/t2d5n8d4x7/1.

*GLP-1*, Glucagon-like peptide-1; *HS*, hidradenitis suppurativa; *SMD*, standardized mean difference.

**Table II:** Substance use disorder incidence and hazard ratios in patients with HS initiating GLP-1 agonists vs metformin

Cohort	Outcome	GLP-1 agonists, *n* (%)	Metformin, *n* (%)	HR (95% CI)	*P*-value
Primary analysis (3-y)	Nicotine dependence	254 (3.37)	323 (4.28)	0.73 (0.62-0.86)	<.001
	Other substance use disorders∗	122 (1.62)	154 (2.04)	0.74 (0.58-0.93)	.011
Sensitivity analysis (5-y)	Nicotine dependence	297 (3.94)	370 (4.90)	0.77 (0.66-0.89)	<.001
	Other substance use disorders∗	141 (1.87)	183 (2.43)	0.74 (0.59-0.92)	.007

Percentages indicate observed event rates. Cox proportional hazards models estimate hazard ratios (HRs) and 95% confidence intervals (CIs). *P*-values are shown unadjusted and remain significant after Bonferroni correction for *P*< .025 for the primary analysis. ICD-10 codes used to define outcomes are listed in Supplementary Table I, available via Mendeley at https://data.mendeley.com/datasets/t2d5n8d4x7/1.

∗ Including alcohol, cannabis, cocaine, and stimulants.

We found that GLP-1 agonists were associated with reduced incidence of nicotine dependence and other substance use disorders in patients with HS, which may be particularly relevant given the high smoking burden in this population. For example, a meta-analysis of 101,977 patients with HS patients reported markedly higher odds of smoking in HS compared with controls (OR = 4.26; 95% CI: 3.10-5.84),^2^ and a longitudinal cohort of 6-million patients found that smoking cessation vs sustained smoking was associated with reduced HS risk (HR = 0.68; 95% CI: 0.56-0.83).^3^

GLP-1 receptor agonists represent a clinically relevant therapeutic class for patients with HS, since they are widely prescribed for metabolic comorbidities in this population. Preclinical and translational studies have suggested that GLP-1 signaling may influence addictive behaviors, including substance use.^1^ Furthermore, in a nationwide cohort of 222,942 diabetic patients, semaglutide treatment was associated with lower tobacco use disorder-related health encounters (HR = 0.68; 95% CI: 0.63-0.74).^4^ Our study extends these findings to patients with HS using a new-user, active-comparator design.

Limitations include reliance on diagnostic coding, including ICD-10 F17 for nicotine dependence, which may introduce misclassification, differential documentation and ascertainment, and residual confounding from uncaptured behavioral, socioeconomic, disease severity, and treatment-selection factors. These limitations could partially explain the observed association.

In sum, we found that GLP-1 agonists were associated with a lower incidence of nicotine and other substance use disorders in patients with HS. Given the central role of smoking for HS risk, severity, and outcomes, these findings warrant further study in this population. Prospective studies incorporating standardized measures of substance use and HS severity are recommended.

### Declaration of generative AI and AI-assisted technologies in the writing process

AI was not used in the preparation of this work.

## Conflicts of interest

Dr Lipner has served as consultant for Moberg Pharmaceuticals and BelleTorus Corporation. Golbasi has no conflicts of interest to declare.
